# Effects of multi-ingredient supplementation on resistance training in young males

**DOI:** 10.2478/v10078-012-0048-y

**Published:** 2012-07-04

**Authors:** Mark ET Willems, Chris W Sallis, Jonathan A Haskell

**Affiliations:** 1 University of Chichester, Department of Sport and Exercise Sciences, College Lane, Chichester, United Kingdom.

**Keywords:** sports nutrition, Cyclone, muscle strength, resistance training

## Abstract

Muscle strength and fatigue resistance increases with resistance training. Resistance training adaptations can be enhanced with single-ingredient or dual-ingredient supplementation but less is known about resistance training adaptations by multi-ingredient supplementation. We examined the effects of a commercial multi-ingredient supplement on resistance training adaptations for training-specific and non-training-specific tasks in young males. Male participants (n = 16, age 21±2 years, body mass 74.5±5.9 kg, body height 177±5 cm) had at least 1 year experience with resistance training exercises. Training (7 muscle groups, 4 sessions/week, weekly adjustments) consisted of two 6 weeks blocks with 4 weeks between blocks. During training, participants consumed placebo (i.e. maltodextrin, n = 7) or the sports nutritional supplement Cyclone (Maximuscle Ltd, UK, n = 9) (main ingredients creatine monohydrate, whey protein, glutamine and HMB) twice daily with one intake <15 min following a training session. Unpaired Student’s t-test was used for placebo and Cyclone group comparison of percentage changes with p < 0.05. Effect sizes (Cohen’s d) were calculated for the Cyclone group. Cyclone did not enhance maximal voluntary isometric force (MVIF) (p = 0.56), time to fatigue at 70% MVIF (p = 0.41) and peak concentric strength (60°·s^−1^) (p = 0.66) of m.quadriceps femoris (i.e. the non-specific training tasks). For the specific-training tasks, Cyclone did not enhance one-repetition maximum (1-RM) of lateral pull (p = 0.48) but there was a trend and large effect size for 1-RM of bench press (p = 0.07, d = 0.98) and 45° leg press (p = 0.07, d = 1.41). Cyclone resulted in an increase in number of repetitions for 80% pre-training 1-RM for lateral pull (p = 0.02, d = 1.30), bench press (p = 0.03, d = 1.20) with a trend for 45° leg press (p = 0.08, d = 0.96). Cyclone during resistance training enhanced the performance of 1-RM and number of repetitions at 80% of pre-training 1RM of some training-specific tasks, all with large effect sizes. Our observations suggest that Cyclone during resistance training substantially improves the ability to perform training-related tasks.

## Introduction

Exercise training programs that incorporate overload with the principles of frequency, intensity and duration result in muscle adaptations and improved performance (Deschenes and Kraemer, 2002; Kubukeli et al., 2002). Adaptations and performance effects are largely specific to the training exercises (Aagaard et al., 1994; Campos et al., 2002; Rutherford and Jones, 1986). For example, an increase in muscle strength is commonly achieved with resistance training programs with learning and coordination effects specifically for movements exerted during training (Rutherford and Jones, 1986). Although a change in muscle function such as muscle strength and endurance are generally the primary goals for such training programs, additional effects include a decrease in fat mass (Hartman et al., 2006), improved muscle metabolism (Goreham et al., 1999), muscle hypertrophy (Mayhew et al., 2009) and lateral force transmission (Erskine et al., 2011).

In an attempt to optimize adaptations to resistance training, use of ergogenic aids such as creatine monohydrate are widespread. Evidence on the effectiveness of supplementation has been obtained mostly in studies examining a single supplement. There is substantial support for the effectiveness of creatine monohydrate to enhance resistance training adaptations (Vandenberghe et al., 1997) potentially by amplification of satellite cells and myonuclei concentration in human skeletal muscle fibres (Olsen et al., 2006). Creatine use during resistance training has also been shown to increase intramuscular IGF-I (Burke et al., 2008), which is part of the signaling pathway to increase muscle mass. Furthermore, creatine use resulted in enhanced functional effects on resistance-training adaptations such as total lifting volume and muscular endurance (Earnest et al., 1995) with some evidence on effects of endurance capacity during sustained isometric contractions (Maganaris and Maughan, 1998). Resistance training stimulates the process of protein turnover in order to enhance performance capacity and stimulate muscle protein accretion, to augment both the neurogenic and myogenic skeletal-muscle adaptive responses (Van Loon, 2007). Indeed, other single supplementation studies have shown that protein supplementation can increase the gain in muscle mass from a resistance training program (Hulmi et al., 2009). The beneficial effect of protein supplementation potentially enhances recovery from the exercise sessions (Hoffman et al., 2010). In addition, whey protein supplementation, in combination with resistance-training also augments the adaptive responses (Beck et al., 2007; Burke et al., 2001; Kerksick et al., 2006). Within the research training literature, it is primarily the effects of single or dual-ingredient supplementation that were studied in conjunction with resistance training.

Less is known on the effectiveness of multi-ingredient supplements on adaptations by resistance training programs (Beck et al., 2007; Kraemer et al., 2007). One study by Chromiak et al. (2004) reported that a 10-wk strength training program combined with a post-exercise recovery drink containing creatine, whey protein, amino acids and carbohydrate, did not provide additional benefits although large variability of the measurements may have lowered statistical power. In addition, the amount of essential elements per serving (3 g of creatine) in that study was relatively small compared to other studies and may not have been sufficient to elevate muscle creatine levels (Chromiak et al., 2004). However, the potential for ingredients in a multi-ingredient supplementation to have synergistic effects would justify the amounts of essential elements to be different than that used as a single supplement. In addition, the establishment of the effectiveness of commercially available multi-ingredient supplementation on the adaptation from resistance training performance may be important for athletic populations.

The purpose of the present study was to examine the effect of a commercial multi-ingredient supplementation (i.e. Maximuscle Cyclone) on the performance of resistance training specific and non-training specific tasks. The resistance training-specific tasks incorporated dynamic movements with single or repetitive lifting that were part of the resistance training program. The main ingredients of Cyclone are creatine monohydrate, whey protein, glutamine and HMB. Therefore, it was hypothesized that the multi-ingredient supplementation Cyclone during resistance training would enhance the performance of training-specific tasks.

## Material and Methods

Twenty-one male participants of the University student population volunteered to take part in the study. Participants had at least one year of experience with resistance training exercises and did not use supplementation for 6 weeks before enrolling in the study. Participants provided informed written consent and no health issues were identified that would prevent them from the safe execution of all required training and functional testing exercises. Familiarization occurred for all training-specific and functional testing exercises. Participants were instructed to abstain from any unaccustomed physical activity for 72 hr and not to do any exercise 24 hr before functional testing and adherence to this instruction was verbally confirmed before each session. Intake of the supplementation and completion of scheduled exercise training sessions were recorded by the participants. When the adherence to supplementation and completion of scheduled exercise training sessions was below 80%, data was excluded from the analysis. This was the case for 5 participants. Characteristics of the remaining 16 participants were age: 21±2 yr (mean±SD), body height: 177±5 cm, body mass: 74.5±5.9 kg, and BMI: 23.6±1.6 kg·m^−2^, respectively. The study used a double-blind randomised protocol. Supplementation was distributed in weekly amounts in unlabeled containers. Supplements were supplied in a powdered form and participants were instructed to consume it by mixing the correct quantity with approximately 250ml of water using unmarked shaker bottles. Maximuscle Cyclone is a multi-ingredient supplementation containing dextrose, whey protein, creatine monohydrate, glutamine, HMB, potassium bicarbonate, sodium bicarbonate, beta-ecdysterone, bioperine and chromium picolinate). Participants were randomized to a Cyclone supplementation group (*n*=9) or placebo group (*n*=7). Participants in the supplementation group consumed daily 2 × 60g of Cyclone (orange flavour, major ingredients per 60g: whey protein: 30g, creatine monohydrate: 5.1g, glutamine 5.1g, and HMB 1.5g) or placebo (maltodextrin), first dose in the morning and second dose within 15 min post-resistance training. On non-training days, supplementation or placebo were taken at similar time points. The amount of Cyclone was not normalized to body weight but based on product advice ensuring ecological validity of this aspect of the study. In addition, the supplementation was self-administrated and participants received weekly the required amount. Performance of training-specific and non-training specific tasks were measured pre and post training. All procedures were approved by the University of Chichester Ethics Committee.

### Resistance Training

Participants followed a high intensity resistance-training program for 12-weeks. The program was divided into two 6-week training blocks and separated by a 4-week phase due to holiday closure of the training facilities. The training program started on the first day of supplementation, incorporated resistance exercise of seven major regions and consisted of 4 training sessions per week. A 3-week training cycle was used with alteration of the muscle groups that were trained to allow appropriate recovery time. The seven regions were chest, back, triceps, biceps, legs, shoulders and abdominals. Regions were grouped into three individual training sessions: 1) chest, triceps, and abdominals, 2) back, shoulders, and abdominals, and 3) legs and biceps. For each exercise, 4 sets were performed for each in a pyramid format. Repetition number decreased from 12, by 2 repetitions per set, to 6, whereas load increased from 70% 1-RM, by approximately 5% per set, to 85% 1-RM. Rest between sets was 1–2 minutes. All participants trained in a fitness facility with at least one partner with weekly load adjustments to complete the required number of repetitions. Adherence in each 6 week training block was required to be above 80% for inclusion in final data analysis. During the 4-week phase between training blocks, participants were instructed not to change dietary habits or normal non-resistance training related physical activity levels. During the 4-week phase, participants did not consume supplementation or placebo.

### Training-specific tasks

One-repetition maximum (1-RM) was determined for the bilateral bench press (BP), 45° leg press (LP) and lateral pull (LAT). All 1-RM testing was performed according to methodology by Burke et al. (2001). Following a warm-up, participants selected a load that would be perceived to complete three repetitions but completed only 1 movement with this load. Load increments ranged from 1.25 to 5.0 kg with a recovery period of two min between attempts. 1-RM was usually reached within 6 attempts. For BP, an Olympic barbell (20 kg) and a free flat bench were used. For this test, participants had their legs approximately shoulder width apart with feet flat and parallel on the floor. Hands were positioned on the bar 10–15 cm from the mounts with an over hand grip, and no arching of the back or buttocks was permitted. For one complete repetition, participants were instructed to lower the bar from the extended arm position to the chest before return movement to the extended arm position. For 1-RM of LP, participants were seated with hands across the chest and feet placed shoulder width and parallel on the plate. Participants lowered the plate from the top extended position, until a knee angle of 45° (approximated by the test administrator), before return to the extended position. For 1-RM of LAT, participants held an overhand grip with vertical back position. Participants lowered the bar from the upright extended position to the chin before return to the upright extended position. A warm-up for all training-specific tasks consisted of 10 repetitions with a self-selected comfortable weight followed by 2 static stretches of 8–10 seconds. Pre- and post-training, number of repetitions to failure at 80% of pre-training 1-RM for BP, LP, and LAT were determined.

### Non-training specific tasks

Peak isokinetic torque was determined for knee extensors muscles of the dominant leg at 60°/s with movement from 90° flexion to 30° extension (Humac®/Norm™, Model 770, CSMi, Inc, USA). Knee axis was aligned with the axis of rotation of the dynamometer and upper body of participants and the lower thigh secured during testing with belts and strap. Participants performed five submaximal contractions before 3 maximal efforts, interspersed by 2 min. Participants had their arms crossed over the chest during all torque testing. Maximal voluntary isometric force of dominant knee extensor muscles was measured with participants seated in a custom-built chair with the upper body firmly restrained against the back of the chair using strap belts over chest and lower thigh. Both hip and knee joint angles were kept at 90° (full extension is 0°). Participants had their arms crossed over the chest during all force testing. The ankle of the participant was connected with a chain to a calibrated stainless steel force transducer (model 616, Tedea-Huntleigh, Cardiff, UK) with a maximum of 2500 N. Real-time force recorded by the force transducer was displayed using Chart for Windows (v. 3.4.1) on a computer screen positioned about 1.5 m in front of the subject, sampled and stored with a frequency of 1000 Hz. Subjects performed three warm-up contractions (duration 4–6 s) with a rest time of 1-min at an estimated intensity of about 30% of the force during maximal voluntary isometric contractions (MVIF), based on familiarization data. Then, subjects were instructed to perform three maximal voluntary isometric contractions (MVIF) for about 2–4 s with a recovery time of 2-min between trials. Participants were encouraged to exceed the maximum force value visible on the computer screen. Strong verbal encouragement was provided during MVIF. Following the MVIF, participants performed a 70% MVIF until task failure. The target force of 70% MVIF was shown as a narrow horizontal bar in the middle of the screen. Subjects received strong verbal encouragement to keep the force signal visible within the screen for as long as possible. Each 70% MIVF was stopped by the experimenter when the force signal became lower than 2.5% of the target force for more than 2 s. Twenty seconds on completion of the isometric fatigue task, each subject performed one final MVIF for determination of fatigue index. Fatigue index induced by the sustained isometric contraction was quantified by the ratio (per cent) of the force value 20 seconds after completion of the isometric fatigue task to the maximal force value of the MVIF before the isometric fatigue task [(i.e. 100% − (MVIF_20s_/MVIF_pre_ × 100%)].

### Dietary Intake

Participants were advised to consume their designated supplement in addition to their normal dietary intake. Participants were required to provide a detailed record of dietary intake during a typical week within the resistance-training program. Data was analysed (CompEat Pro, Nutrition Systems, Banbury, England) for calculation of a weekly energy intake (kcal).

### Statistical Analysis

Data were reported as mean±SD. Percentage changes of test parameters from baseline were calculated for placebo and Cyclone and analysed with unpaired Student t-test using SPSS for Windows version 16.0 software. Effect sizes (Cohen’s *d*) were calculated for the Cyclone group. Significance was set at an alpha level of p≤0.05.

## Results

The weekly caloric intake was similar for the Placebo (10991 ± 4211 kcal) and the Cyclone group (11712± 6671 kcal). Placebo and Cyclone group completed 95% and 91% and 96% and 89%, respectively, of scheduled training and supplementation intake requirements. Changes in body mass were similar (Placebo: 1.8±1.6%, Cyclone: 2.1±1.8%) (*p* = 0.45).

### Training-specific tasks – muscle strength

**Figure 1:**
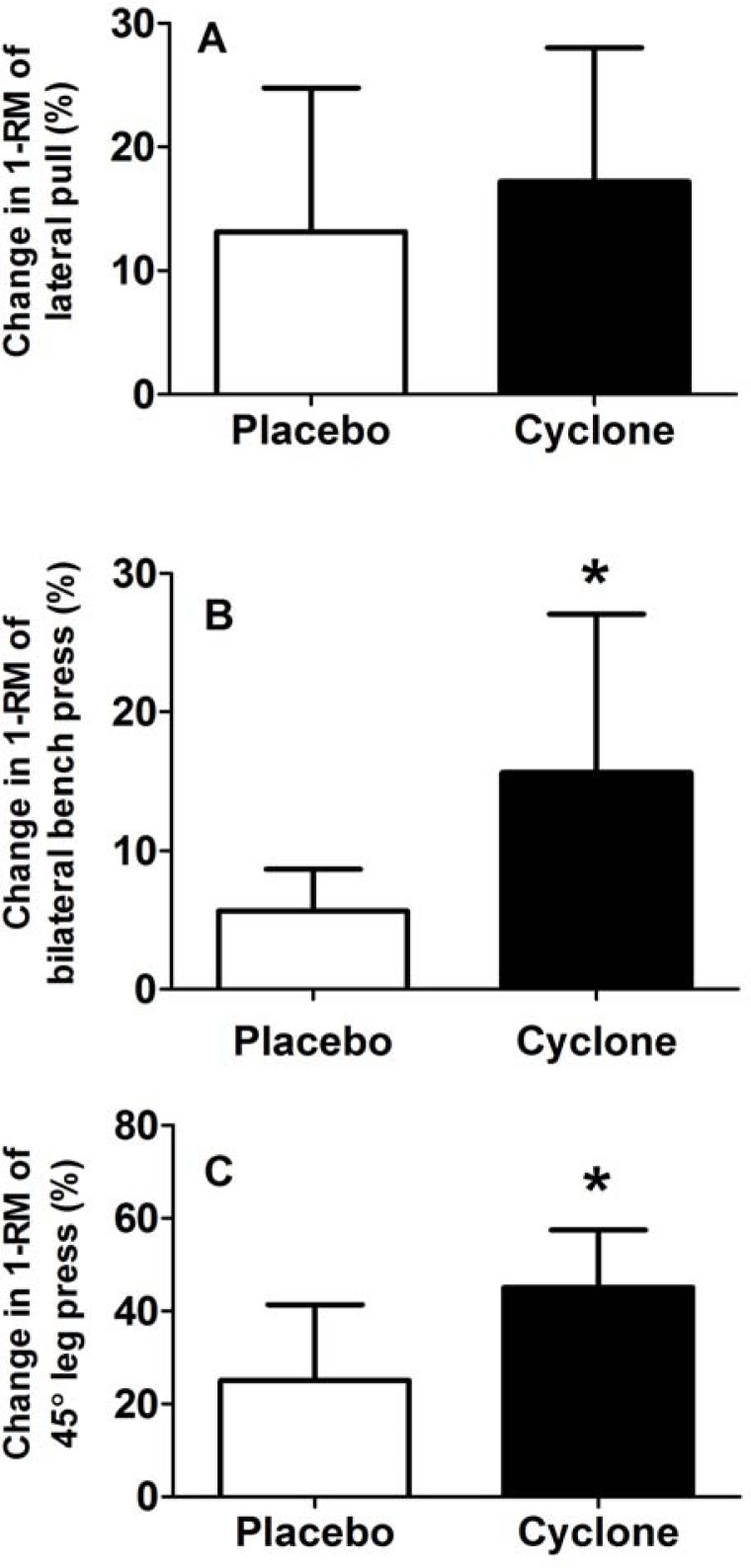
Changes in 1-RM of lateral pull (A), bilateral bench press (B) and 45° leg press (C) in response to a resistance training program with placebo or Cyclone.

Cyclone did not enhance one-repetition maximum (1-RM) of lateral pull (Placebo: 13±12%, baseline: 72±9 kg; Cyclone: 17±11%, baseline: 68±13 kg) (*p* = 0.48) (Figure 1A) but there was a trend with large effect sizes for 1-RM of bilateral bench press (Placebo: 6±8%, baseline: 86±17 kg; Cyclone: 16±11%, baseline: 80±25 kg) (*p* = 0.07, *d* = 0.98) (Figure 1B) and 45° leg press (Placebo: 25±16%, baseline: 343±66 kg; Cyclone: 45±12%, baseline: 314±55 kg) (*p* = 0.07, *d* = 1.41) (Figure 1C).

### Training-specific tasks – muscle endurance

**Figure 2:**
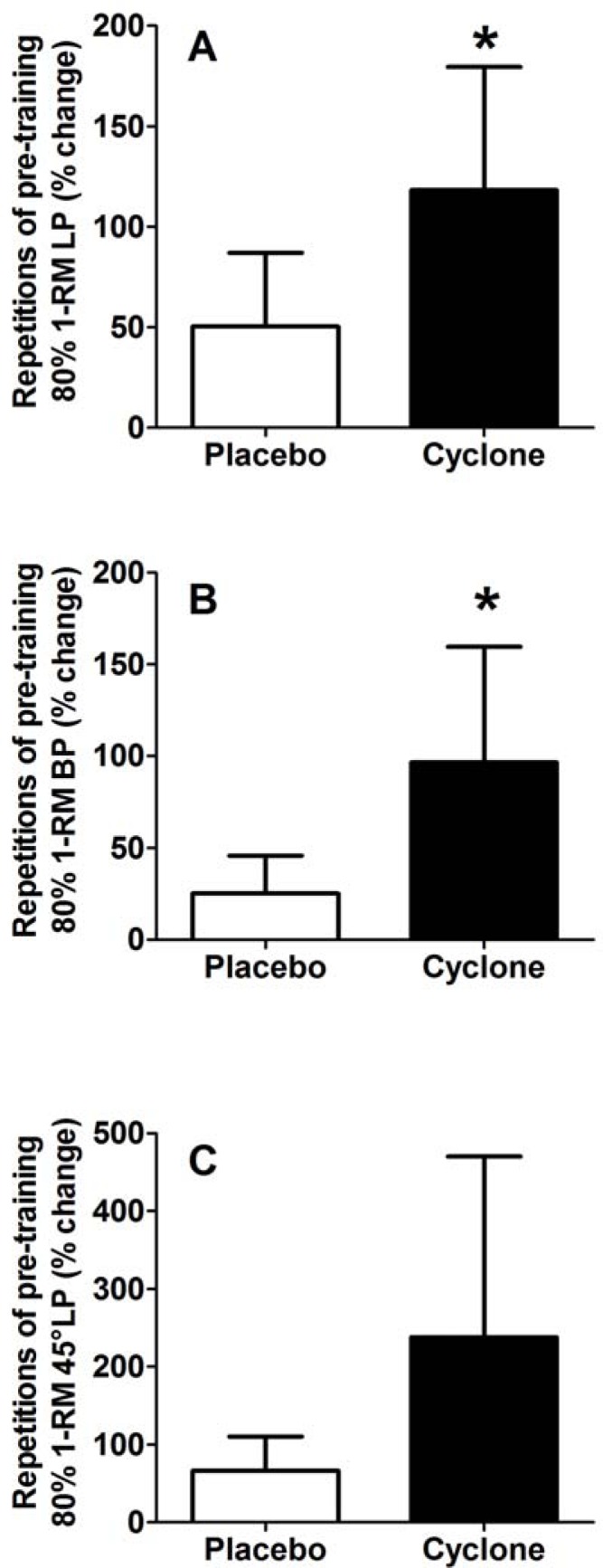
Changes in repetitions of pre-training 80% 1-RM for lateral pull (LP) (A), bilateral bench press (BP) (B) and 45° leg press (45°LP) (C) in response to a resistance training program with placebo or Cyclone.

Cyclone resulted in an increase in number of repetitions for 80% pre-training 1-RM with large effect sizes for lateral pull (Placebo: 50±37%, baseline: 13±3; Cyclone: 118±61%, baseline: 11±3) (*p* = 0.02, *d =* 1.30) (Figure 2A), bilateral bench press (Placebo: 25±55%, baseline: 8±4; Cyclone: 96±63%, baseline: 8±3) (*p* = 0.03, *d =* 1.20) (Figure 2B) and a trend for 45° leg press (Placebo: 67±44%, baseline: 13±5; Cyclone: 238±232%, baseline: 14±6) (*p* = 0.08, *d =* 0.96) (Figure 2C).

### Non-training-specific tasks

Cyclone did not enhance maximal voluntary isometric force (MVIF) (Placebo: −4±5%, baseline: 742±45 N; Cyclone: −2±8%, baseline: 743±117 N) (*p* = 0.56), time to fatigue at 70% MVIF (Placebo: 12±25%, baseline: 41.2±8.3 s; Cyclone: 27±40%, baseline: 35.4±8.3 s) (*p* = 0.41), fatigue index from a 70%MVIF (Placebo: −26±31%, baseline: 20.5±5.0%; Cyclone: −4±31%, baseline: 20.0±3.7% (*p* = 0.38), and peak concentric strength (60°·s^−1^) (Placebo: 5±17%, baseline: 211±24 N·m; Cyclone: 9±20%, baseline: 202±34 N·m) (p = 0.66) of *m.quadriceps femoris*.

## Discussion

The observations from the present study on the use of Cyclone during resistance training demonstrated a significant enhanced effect on the strength and strength-endurance for some training-specific tasks. Some of the training-specific tasks showed a trend towards improvement in favour of the supplement, the trend was associated with large effect sizes. The association between trend and effect size is worth noting as the relatively small sample size in our study would then suggest the potential for credible effectiveness of the multi-ingredient supplement Cyclone on all training-specific tasks of strength and muscular endurance. At baseline, values for strength and fatigue were similar between the placebo and Cyclone group (*t*-test, p>0.05). In addition, strength values for some of the training specific tasks in our study at baseline in comparison to values reported in other studies were comparable [bench press: ∼81 kg (Walter et al., 2009), ∼108 kg (Roberts et al., 2007), ∼75 kg (Dolezal and Potteiger, 1998) or different (leg press: ∼215 kg (Walter et al., 2009), ∼392 kg (Roberts et al., 2007). At baseline, our number of repetitions was similar to those of Beck et al. (2007) for bench press (7.5±2.3 repetitions) and leg press (13.8±5.4 repetitions). Therefore, our large effect sizes for strength and strength-endurance measurements were not due to low baseline values compared to other resistance training studies.

Differences for ingredients and dosage in studies of the effects of multi-ingredient use during resistance training studies and characteristics of the training programme and participants complicate a comparison with the effects of other studies. However, as far as we know, most multi-ingredient supplementation used in resistance training studies contain creatine (Beck et al., 2007; Kraemer et al., 2007; Schmitz et al., 2010; Shelmadine et al., 2009). There is strong support that creatine supplementation during resistance training is more effective in enhancing training specific task for strength and strength-endurance than resistance training alone (Rawson et al., 2003). Studies on creatine and resistance training used a short duration high dose intake followed by a low dose maintenance (e.g. 20 g/d for 4 days followed by 5 g/d for the time period of the study, Vandenberghe et al., 1997) or amount of creatine similar to our study. In our study, the daily dose of creatine monohydrate (i.e. 2 × 5.1 g) was higher than those used in most other multi-ingredient studies (5.0 g (Beck et al., 2007), 3.0 g (citrate and monohydrate) (Kraemer et al., 2007), 4 g (Schmitz et al., 2010)). The amount of creatine intake was not specified in the study by Shelmadine et al. (2009) on the effects by SOmaxP. Our participants took the supplements on all training and non-training days within the 6 weeks blocks which is similar (Beck et al., 2007; Kraemer et al., 2007; Shelmadine et al., 2009) or different to other studies (only on training days, Schmitz et al., 2010). Creatine monohydrate supplementation is known to benefit the performance of repeated bouts of high intensity exercise as increased content of phosphocreatine may enhance ATP resynthesis (Volek and Kraemer, 1996). It is therefore possible that the training-induced adaptations by Cyclone were due to an ability for the participants to train with larger volumes and intensity (Casey et al., 1996). Such training parameters were not controlled or recorded in our study. Differences in training progression may have happened and are essential for understanding of the enhanced effect by supplementation (Syrotuik et al., 2000). It is likely that the amount of creatine in Cyclone resulted in elevation of phosphocreatine levels in responders in our study. Future studies on the effectiveness of multi-ingredient supplementation and resistance training should take into account the effect of differences in progression of training.

Other components of Cyclone (i.e. whey protein and HMB) have been shown to be beneficial in single supplementation studies and resistance training exercise. Whey is a protein form that contains a higher concentration of essential amino acids in comparison to other protein sources. It possesses high biological value and rapid absorption kinetics (Pennings et al., 2011). Whey protein has been suggested to enhance muscle cell repair following resistance exercise and synthesis of protein elements essential for muscular hypertrophy (Beck et al., 2007). Kerksick et al. (2007) suggested that intensive resistance-training reduces the availability of essential amino acids, which in turn, may decrease the rate of tissue repair and growth. Ingestion of whey protein via post training supplementation would subsequently generate a rapid increase in the plasma volume levels of amino acids, producing elevated protein synthesis, and little change in protein catabolism (Kerksick et al., 2006). Whey protein supplementation is purported to elicit a higher blood amino acid peak and prevent protein degradation (Kerksick et al., 2007). The amount of whey protein in our study (i.e. 60 g/d) was higher compared to other studies on multi-ingredient supplementation and resistance training (13 g serving (Chromiak et al., 2004); 7 g serving (Schmitz et al., 2010) or comparable (Burke et al., 2001)). In that study, Burke et al. (2001) found no effect on knee flexion peak torque, 1-RM for the bench press and squat exercises were unaffected. The amount of HMB in our study (3 g/d) was similar to the study by Panton et al. (2000). HMB is a metabolite of the essential amino acid leucine. It may enhance gains in strength associated with resistance training (Slater and Jenkins, 2000). HMB has been suggested to act as an anti-catabolic agent, minimizing protein degradation, and muscular cell damage as a result of high-intensity resistance-training, stimulating increased gains in strength. It was reported that short-term HMB supplementation during resistance training significantly enhanced upper body strength (Panton et al., 2000). Not all research supports gains in muscular function with HMB supplementation (for a review see Wilson et al., 2008). During 4-weeks of HMB supplementation, in comparison to a placebo, no significant changes in strength, expressed as gains in total weight lifted in a maximal repetition test at a load equal to 70% of 1RM, for the BP, squat, and power clean exercises were reported (Kreider et al., 1997). It was concluded that HMB supplementation during training provides no ergogenic value to experienced resistance-trained athletes (Kreider et al., 1997). Although our groups had at least one year of experience with resistance training exercises, our group of participants could not be considered experienced resistance-trained athletes.

Besides creatine monohydrate, whey protein and HMB, Cyclone contains ingredients for which there is no strong evidence to be beneficial for enhancement of strength and/or endurance adaptations by resistance training. Glutamine has been suggested to enhance protein synthesis and minimise catabolic responses during heavy resistance-training, increasing muscular hypertrophy, and reducing exercise-induced immunosuppression (Kreider, 1999) but others reported no effect of glutamine supplementation in combination with a six-week resistance-training program (Candow et al., 2001). A recent review by Phillips (2007) confirmed the lack of evidence for a performance enhancing effect of glutamine. Bicarbonates may have had a buffering effect during training sessions but amounts present in Cyclone are low compared to other studies (Price et al., 2003). Other ingredients such as bioperine are thought to enhance thermogenesis.

It is concluded that supplementation with Cyclone during resistance training enhanced the performance only of training-specific tasks, i.e. 1-RM and number of repetitions at 80% pre-training 1-RM. Our observations suggest that Cyclone during resistance training substantially improves the ability to perform training-related tasks in young adult males.
